# An Enzyme-Catalyzed Multistep DNA Refolding Mechanism in Hairpin Telomere Formation

**DOI:** 10.1371/journal.pbio.1001472

**Published:** 2013-01-29

**Authors:** Ke Shi, Wai Mun Huang, Hideki Aihara

**Affiliations:** 1 Department of Biochemistry, Molecular Biology and Biophysics, University of Minnesota, Minneapolis, Minnesota, United States of America; 2 Department of Pathology, University of Utah Health Sciences Center, Salt Lake City, Utah, United States of America; University of California, Los Angeles, United States of America

## Abstract

Hairpin telomeres of bacterial linear chromosomes are generated by a DNA cutting–rejoining enzyme protelomerase. Protelomerase resolves a concatenated dimer of chromosomes as the last step of chromosome replication, converting a palindromic DNA sequence at the junctions between chromosomes into covalently closed hairpins. The mechanism by which protelomerase transforms a duplex DNA substrate into the hairpin telomeres remains largely unknown. We report here a series of crystal structures of the protelomerase TelA bound to DNA that represent distinct stages along the reaction pathway. The structures suggest that TelA converts a linear duplex substrate into hairpin turns via a transient strand-refolding intermediate that involves DNA-base flipping and wobble base-pairs. The extremely compact di-nucleotide hairpin structure of the product is fully stabilized by TelA prior to strand ligation, which drives the reaction to completion. The enzyme-catalyzed, multistep strand refolding is a novel mechanism in DNA rearrangement reactions.

## Abstract 2

Crystal structures reveal catalysis of DNA refolding in the molecular mechanism underlying generation of bacterial hairpin telomeres.

## Author Summary

Linear chromosomes capped by hairpin telomeres are widespread in prokaryotes and are found in important bacterial pathogens. However, three-dimensional structure of the hairpin telomere, as well as the molecular mechanisms underlying its generation, has remained poorly understood. In this work, we investigated how the enzyme responsible for generating the bacterial hairpin telomeres (protelomerase, also known as telomere resolvase) transforms a linear double-stranded DNA molecule into sharp hairpin turns. Our X-ray crystallographic and biochemical data collectively suggest that protelomerase employs a multistep DNA strand-refolding mechanism as described below. Protelomerase first cleaves both strands of a double-helical DNA substrate and reshapes the DNA strands into a transition state conformation (refolding intermediate) stabilized by specific protein–DNA and DNA–DNA interactions including noncanonical (non-Watson–Crick) base-pairs. The DNA strands are then refolded into extremely compact hairpin products, stabilized by a set of interactions distinct from those stabilizing the refolding intermediate. We believe that an enzyme “catalyzing” not only the chemical reactions of DNA strand cutting/rejoining but also the ordered transition between different DNA conformations to guide refolding of the DNA strand is a novel concept, and we suspect that similar mechanisms may be employed by other enzymes involved in conformational changes/refolding of biological macromolecules.

## Introduction

**Figure 1:**
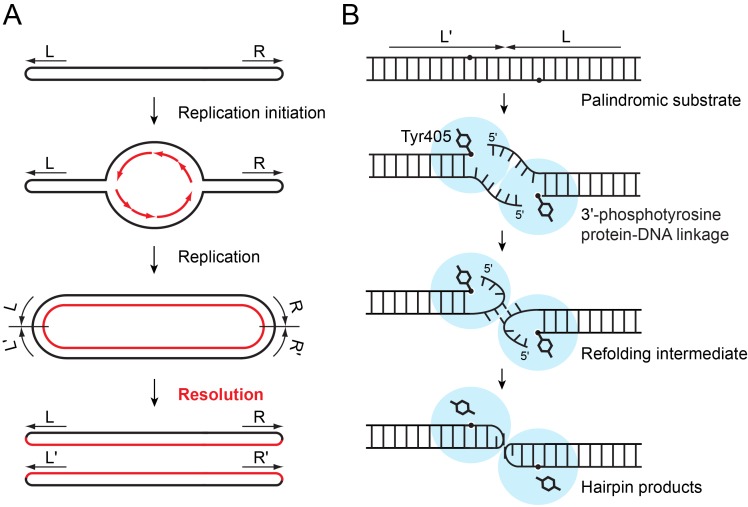
Hairpin telomere generation by protelomerase TelA. (A) Schematic diagram showing how a bacterial linear chromosome with hairpin telomeres is replicated [6],[7],[10]. The last step is resolution of concatenated chromosomes at the inverted repeat junctions, which is carried out by protelomerase (also known as telomere resolvase). (B) A model for the hairpin telomere formation by the protelomerase TelA, based on the crystal structures presented in this study. A key feature of the reaction mechanism of TelA revealed by this study is the “refolding intermediate” conformation that is stabilized by a set of transient protein–DNA and DNA–DNA interactions.

Telomeres at the termini of linear chromosomes protect the DNA ends from degradation or aberrant repair reactions including end-fusion, while allowing complete replication of the terminal sequences [1]. The simplest form of telomere is a covalently closed hairpin loop found in bacteria carrying linear chromosomes, including *Borrelia* spirochetes—the causative agents of Lyme disease and relapsing fever [2],[3], *Agrobacterium* [4],[5], and cyanobacteria. Replication of such bacterial linear chromosomes with hairpin telomeres starts from an internal origin and proceeds bi-directionally, yielding a circular intermediate composed of a head-to-head, tail-to-tail dimer of chromosomes (Figure 1A) [6],[7]. The circular dimeric chromosome is then resolved at the two inverted-repeat junctions, formed as replication traverses through the telomeres, by a dedicated DNA cleavage-rejoining enzyme called protelomerase (also known as telomere resolvase) to regenerate unit-length chromosomes with hairpin telomeres [8]–[11].

The protelomerase enzymes function as a dimer, making staggered cleavages of both strands of DNA to form covalent 3′-phosphotyrosine linkages, then exchanging the released 5′-ends while holding onto the 3′-ends, and finally sealing the broken DNA strands to generate two hairpin ends (Figure 1B) [12],[13]. The phosphotyrosine-mediated DNA cleavage-rejoining reaction is chemically isoenergetic with no intrinsic directional bias, in a similar fashion to the reactions catalyzed by topoisomerases [14] and tyrosine-recombinases [15] that interconvert between linear double-helical DNA substrates and products. However, protelomerase is unique in that it converts the canonical duplex conformation of DNA into strained hairpin structures. Although the crystal structure of a bacteriophage-derived protelomerase TelK bound to a linear duplex substrate DNA was reported (Figure S3, top panel) [16], the TelK–DNA complex structure provided little information regarding events following the strand cleavages or structure of the hairpin telomere products. Thus, mechanisms by which protelomerase drives the hairpin formation reaction forward without an exogenous input of energy or getting trapped in a DNA cleavage–religation equilibrium are not well understood.

We describe here a series of crystal structures of a bacterial protelomerase bound to reaction intermediates and hairpin products, which reveal that the enzyme dimer actively stabilizes both the tightly folded hairpin products and a transition state of DNA refolding pathway following the DNA strand cleavages. Furthermore, we show that the hairpin telomere formation by protelomerase is highly sequence-dependent, in line with the multistep strand-refolding mechanism suggested by the crystal structures. Thus, our collective results suggest that protelomerase catalyzes not only the chemical reactions of DNA strand cutting and rejoining but also the ordered transition between different DNA conformations to guide refolding of a DNA strand. Since DNA hairpins are formed as key intermediates in transposition [17]–[20] and the use of transposon-type motif by the *Borrelia* protelomerase/telomere resolvase ResT has been reported [21], our findings on protelomerase could provide insights into the mechanisms of transposon-related DNA remodeling reactions, including the V(D)J recombination responsible for diversifying antigen-receptor genes in higher vertebrates [22]–[24].

## Results and Discussion

### Overall Structure of the TelA–DNA Complex

**Figure 2:**
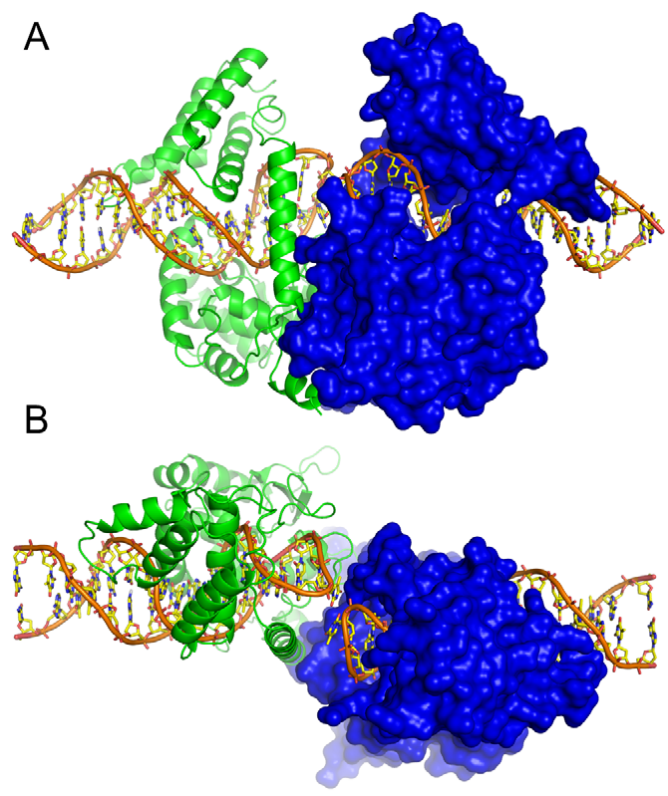
Overall structure of the TelA dimer bound to hairpin telomeres. Molecular surface is shown for one of the molecules in the TelA dimer, whereas the other molecule is shown by ribbons. The view in (A) is perpendicular to and that in (B) is parallel to the 2-fold axis relating the two TelA molecules. The two hairpin DNAs in alternative conformations are shown, as in Figure 4E.

To better understand how protelomerase functions, we have crystallized the full-length protelomerase from the plant pathogen *Agrobacterium tumefaciens* C58 (TelA) in complex with DNA substrates containing the terminal sequences of the *Agrobacterium* linear chromosome. We used several types of DNA substrates (Figure S1) resulting in crystal structures of TelA–DNA complexes that differ in the DNA conformation for the region ultimately forming the hairpin turn (shown by red letters in Figure S1). Phases for a parental TelA–DNA complex were obtained by the selenomethionine SAD phasing method, and all crystal structures have been refined to 2.2∼2.4 Å resolution. Due to strongly anisotropic diffraction, the resolution limits for the refinement were set differently along the three principal axes (Table 1). In the crystals, the asymmetric unit contains a single TelA molecule bound to a hairpin telomere sequence, and the crystallographic dyad generates the TelA homodimer responsible for resolution of a replicated hairpin telomere (Figure 2). Our models consist of residues 102 to 421 of the full-length 442 residues TelA protein bound to a half-site DNA substrate consisting of a 13 or 14 bp double-stranded stem and a 5′-overhang (Figure S1). The electron density for the N-terminal ∼100 residues was very weak, likely reflecting high flexibility. This region is not required for in vitro hairpin formation by TelA [5].

**Table 1:** X-ray data collection, phasing, and refinement statistics.

	TelA/DNAa+VO_4_^3−^	R255A/DNAa	TelA/DNAb	SeMet-TelA/DNAc	Y201A/DNAb	R205A/DNAb6	TelA/DNAd
	Hairpin Product	Unligated Hairpin	p-Tyr Complex Trapped with a Nick	p-Tyr Complex (Se) Trapped with a Nick	Mutant Complex	Mutant Complex	p-Tyr Complex Trapped with a Mismatch
Protein Data Bank ID	4E0G	4E0J	4E0P	4DWP	4E10	4E0Z	4E0Y
Beamline	APS 24-ID-C	APS 24-ID-C	APS 14-BM-C	APS 14-ID-B	APS 24-ID-C	APS 24-ID-C	APS 24-ID-C
**Data collection**							
Space group	C2	C2	C2	C2	C2	C2	C2
Cell dimensions							
*a,b,c* (Å)	117.88, 120.20, 58.04	117.56, 120.08, 56.76	117.58, 119.69, 62.94,	117.85, 119.77, 65.98,	116.34, 119.66, 56.63	117.27, 120.39, 58.72	116.98, 119.44, 56.92
*β* (°)	111.85	111.68	113.94	108.63	111.30	112.46	112.42
Wavelength (Å)	0.979	0.979	0.900	0.979	0.979	0.979	0.979
Resolution (Å)	50–2.20 (2.24–2.20)	50–2.30 (2.34–2.30)	50–2.20 (2.24–2.20)	50–2.35 (2.39–2.35)	50–2.40 (2.44–2.40)	50–2.42 (2.46–2.42)	50–2.40 (2.44–2.40)
R*_sym_* (%)	7.8 (38.2)	9.5 (20.0)	6.4 (19.9)	8.3 (23.7)	4.6 (23.2)	7.0 (25.5)	6.7 (34.3)
I/σ(I)	13.5 (4.0)	22.17 (5.15)	22.5 (3.9)	24.4 (5.1)	15.7 (1.6)	15.5 (4.7)	14.1 (3.0)
Completeness (%)	99.2 (98.8)	88.2 (65.2)	94.5 (83.2)	83.6 (52.9)	88.2 (72.6)	87.0 (60.0)	98.8 (98.1)
Redundancy	3.3 (3.1)	3.4 (3.0)	6.4 (5.1)	6.4 (4.5)	2.0 (1.7)	3.2 (2.9)	3.2 (2.9)
**Phasing (25–3.2 Å)**							
Sites				11			
FOM				0.49			
**Refinement**							
Resolution (Å)	50–2.20	50–2.30	50–2.20	50–2.35	50–2.50	50–2.42	50–2.40
Anisotropic resol. a*,b*,c* (Å)	2.2, 2.2, 3.0	2.3, 2.3, 2.7	2.2, 2.2, 3.1	2.35, 2.35, 2.8	2.5, 2.5, 3.2	2.42, 2.42, 3.1	2.4, 2.4, 3.2
Number of reflections	30,350	27,714	32,055	29,403	18,263	25,089	21,996
R_work_/R_free_	19.4/23.8	20.5/25.2	20.1/24.9	18.9/22.7	19.5/26.8	19.1/25.3	21.6/27.0
Number of atoms	3,683	3,681	3,597	3,542	3,280	3,417	3,316
Macromolecules	3,275	3,313	3,146	3,195	3,101	3,137	3,174
Ligands	3	2	12	—	—	24	6
Water	405	363	439	347	179	256	136
B-factors	37.80	47.40	52.80	40.60	66.40	58.70	59.30
Macromolecules	38.10	47.80	53.40	40.40	67.30	58.30	59.80
Solvent	35.50	43.20	49.30	42.20	49.80	61.00	44.10
R.m.s. deviations							
Bond lengths (Å)	0.011	0.003	0.011	0.010	0.018	0.012	0.003
Bond angles (°)	1.09	0.77	1.49	1.32	1.66	1.58	0.86

Statistics for the highest resolution shell are shown in parentheses.

**Figure 3:**
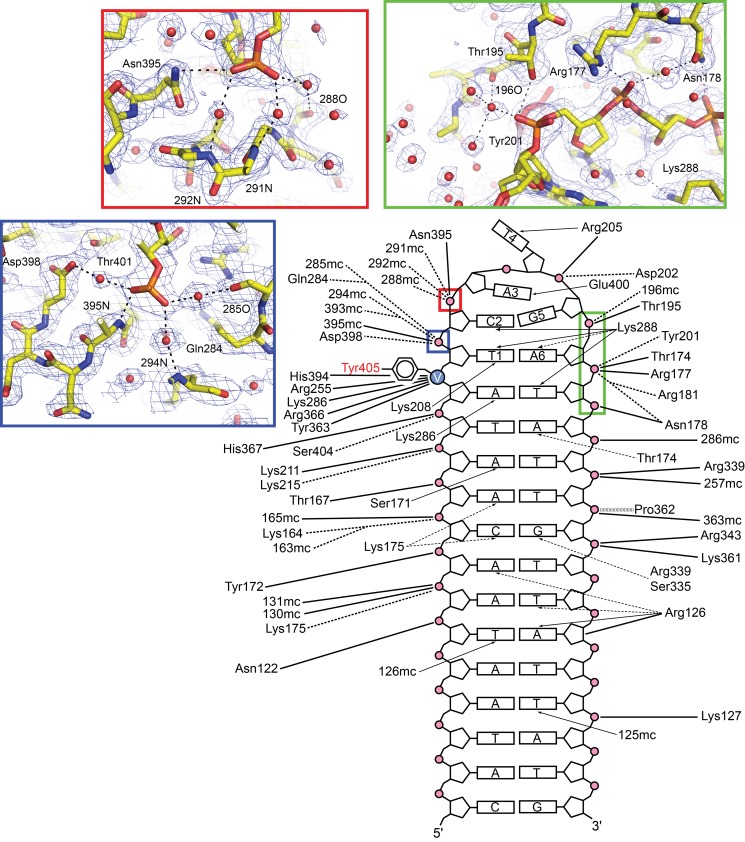
Schematic diagram and close-up views of the TelA–hairpin DNA interactions. Solid lines and dashed lines denote direct and indirect (water-mediated) interactions, respectively. The close-up views in the insets show interactions involving the phosphate backbones in the hairpin loop. The 2Fo-Fc electron densities are contoured at 1.5 σ.

The TelA monomer consists of two structural domains, the catalytic domain and an α-helical bundle domain, that together clamp down the DNA substrate (Figure 2). While TelA shows significant sequence homology to the bacteriophage-derived protelomerase TelK [16],[25] only in the ∼190-residue region of the catalytic domain including the active site residues (∼25% identity), the three-dimensional structure of TelA closely resembles that of the core part of the larger TelK protein (Figure S2). Each of the two domains of TelA interacts with DNA in both the major and minor grooves, with a total of 43 residues positioned in close proximity to DNA (<3.6 Å). Six base-pairs of the binding site are recognized by direct hydrogen bonding interactions, with additional base-pairs involved in water-mediated hydrogen bonds or van der Waals contacts (Figures 3, S4, and S5). The α-helical linker (Ala198–Gly217) connecting the two domains harbors several residues that make DNA contacts. Among these residues, Tyr201 and Arg205 play key roles in refolding the duplex DNA substrate into hairpin products as discussed later. The catalytic domains in the TelA dimer interact extensively with one another, burying 1,147 Å^2^ of surface area per subunit. The two protein molecules in the TelA dimer bound to DNA are positioned such that there is a large (>10 Å) offset in the DNA helical axes across the dimer interface, similar to the arrangement observed in the TelK–DNA complex (Figures 2B and S3) [16].

### Hairpin Product Complex

**Figure 4:**
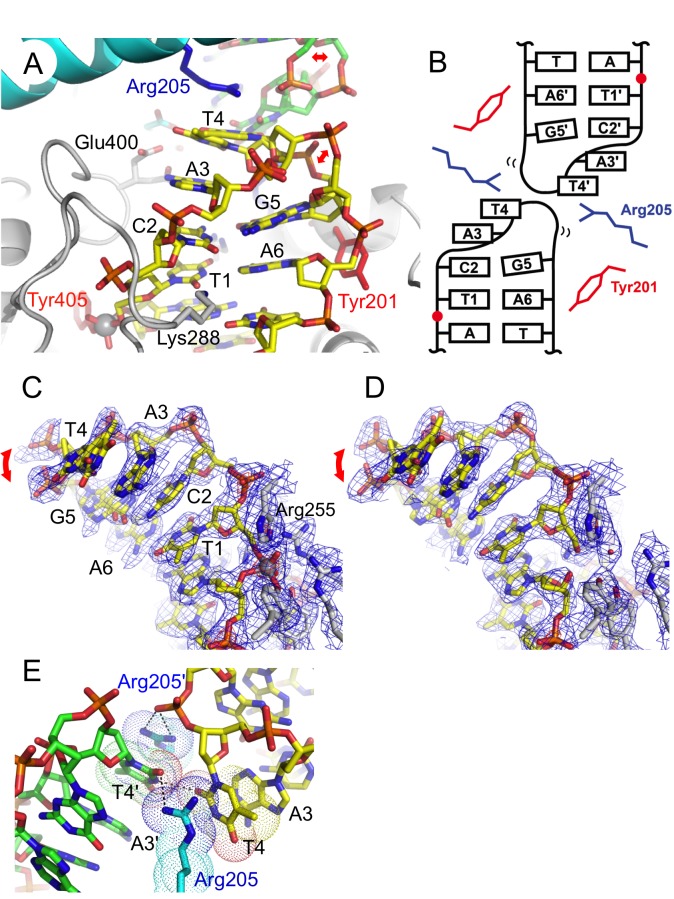
Structure of the hairpin telomere bound to TelA. (A) The compact di-nucleotide hairpin DNA product. All bases except two at the apex (Ade3 and Thy4) form Watson–Crick base-pairs. The DNA strand near the tip of the hairpin loop adopts two alternative conformations as indicated by the red arrows. The two protein subunits and two DNA molecules in the TelA–DNA complex are all colored differently (yellow/green for DNA, and grey/cyan for protein) to highlight *cis* versus *trans* interactions made by TelA. (B) Schematic diagram of the hairpin DNA conformation. The red dots represent the scissile phosphates. (C and D) Simulated annealing composite omit 2Fo-Fc densities for the wild-type TelA–hairpin DNA complex (C) and the R255A unligated hairpin complex (D). Electron densities within 2.0 Å from the DNA or active site protein atoms are shown, contoured at 1.5 σ (blue) or 7.0 σ (red) above the mean levels. (E) Two hairpin ends in alternative conformations are packed tightly across the 2-fold axis, interacting with one another as well as with Arg205. Van der Waals radii for Arg205 and Thy4 are shown by dots.

**Figure 5:**
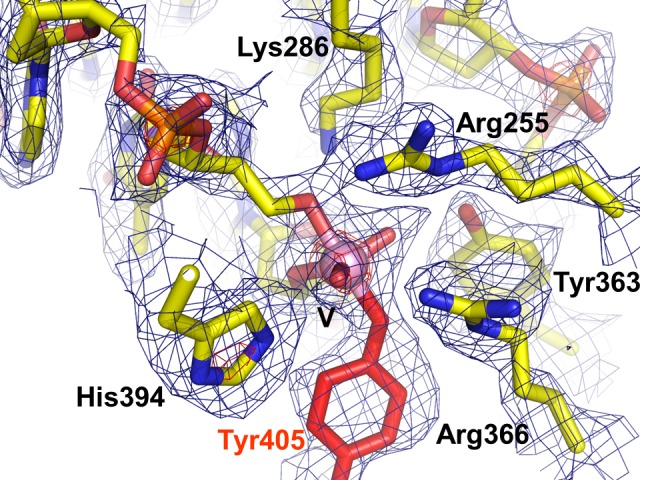
The enzyme active site. Orthovanadate bridges the DNA 5′-OH group, 3′-OH group, and the Tyr405 sidechain in the wild-type TelA hairpin–vanadate complex. The magenta sphere in the middle represents vanadium in the active site. The active site of TelA is similar to those of type-IB topoisomerases and tyrosine recombinases. But TelA is unique in having Tyr at residue 363 (commonly Lys in topoisomerases and His in tyrosine recombinases). The *Borrelia* protelomerase/telomere resolvase ResT also has Tyr at this position [32]. Simulated annealing composite omit 2Fo-Fc densities within 2.0 Å from the DNA or active site protein atoms are shown, contoured at 1.5 σ (blue) or 7.0 σ (red).

By crystallizing the wild-type TelA bound to a palindromic target DNA nicked at the scissile positions 6 bp apart (DNAa in Figure S1) in the presence of orthovanadate, we obtained a TelA dimer bound to the covalently closed hairpin products. The self-complementary six-base overhangs (T_1_C_2_A_3_T_4_G_5_A_6_) with the native sequence of the *Agrobacterium* chromosome terminus form sharp hairpin turns in two alternating conformations, and are packed tightly between the two active sites in the TelA dimer (Figure 4). The vanadate moiety links the 5′-and 3′-OH groups across the DNA nick as well as the Tyr405 nucleophile in the active site, mimicking the pentavalent transition state of the DNA ligation reaction (Figure 5). The DNA strands take an extremely compact hairpin conformation in which all bases except two at the apex form the canonical Watson–Crick base-pairing. The two unpaired bases (Ade3 and Thy4) remain intrahelical and stack on the base-paired stem of the hairpin DNA, with rises per base steps comparable to that in the B-form DNA (∼3.4 Å) (Figure 4A,B).

**Figure 6:**
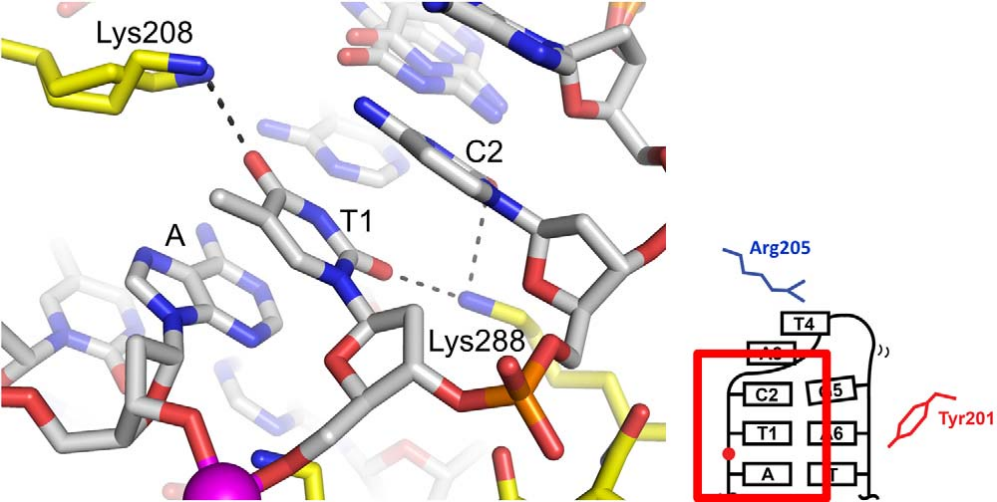
Sequence-specific contacts near the hairpin end. Sequence-specific interactions by Lys208 and Lys288 that stabilize the 5′-terminal Thy1 base in the hairpin product bound to TelA. The magenta sphere in the very bottom of the image represents vanadium in the active site. Two alternative conformations are shown for the Lys208 sidechain.

The compact di-nucleotide hairpin structure is stabilized by TelA through a number of interactions. The guanidinium group of Arg205 makes a cation-π stacking interaction on the thymine base (Thy4) at the tip of the hairpin turn (Figure 4A,E). The stacking interaction by Arg205 is made *in trans*—that is, the interdomain linker contributing Arg205 to cap a hairpin DNA product connects the catalytic and DNA-binding domains clamping down the other hairpin half-site in the TelA dimer. The α-helical interdomain linker segment completely blocks the helical path of each DNA half-site, suggesting that the linear duplex substrate DNA would have to be severely distorted to fit in the DNA-binding path of the TelA dimer. The backbone phosphate group of Cyt2, Ade3, and Ade6 in the hairpin turn each forms both direct and water-mediated hydrogen bonds/salt bridges with TelA (Figure 3). Sequence-specific contacts are made by the carboxyl group of Glu400 that interacts with the unpaired Ade3 base in both of its two alternate sidechain conformers (Figure 4A), Lys208 that hydrogen bonds with the O4 atom of Thy1 in the major groove, and Lys288 that interacts with the O2 atoms of Thy1 and Cyt2 in the minor groove (Figure 6).

Refinement of the atomic model of the TelA-hairpin DNA–vanadate complex at 2.2 Å resolution allowed two alternate conformations of DNA strand in the hairpin turn to be resolved (indicated by the red arrows in Figure 4A and 4C). The backbone phosphate group of Gua5 at the apex of the hairpin turn as well as the flanking bases Thy4 and Gua5 take two distinct positions with approximately equal partial occupancies. The terminal base Thy4 is stacked more closely with Arg205 *in trans* in one conformer, while the backbone phosphate of Gua5 in the other conformer forms a salt bridge with the same Arg205 in *cis*. Thus, Arg205 plays dual roles in stabilizing the tight hairpin turn bound to the TelA dimer. For steric reasons, the two juxtaposed hairpin ends in a TelA dimer must be in alternative conformations, which introduces asymmetry into the otherwise 2-fold symmetric TelA–DNA complex (Figure 4E). The two hairpin ends have backbone phosphorus atoms positioned only 5.9 Å apart across the 2-fold axis (intrastrand phosphorus distance in the B-form DNA is ∼6.8 Å), highlighting the tight packing of hairpin turns in the TelA dimer.

Having revealed the structure of the hairpin telomere, we then asked whether stabilization of the extremely compact DNA hairpin conformation by TelA is dependent on covalent closure of the DNA strand. To address this question, we used TelA with a point mutation in one of the active site residues Arg255 that plays an essential role in coordinating the scissile phosphate (Figure 4C and Figure 5). TelA•R255A was crystallized in complex with the palindromic target DNA sequence nicked at the scissile positions (DNAa in Figure S1). The resulting 2.3 Å resolution crystal structure of the TelA•R255A–DNA complex shows the exact same hairpin DNA conformation as observed in the wild-type TelA–hairpin DNA–vanadate complex described above (r.m.s.d. of 0.41 Å for the six nucleotides in the hairpin), except that the scissile phosphate-binding site is empty and nothing bridges the 5′ and 3′-OH groups of DNA (Figure 4D). As the DNA hairpin region is free from crystal lattice contacts (Figure S6), the result suggests that the hairpin conformation observed in our crystal structures is the most thermodynamically preferred conformation of the telomere DNA sequence when bound to TelA, even in the absence of a covalent phosphodiester linkage. This argues against a model in which strand ligation captures the hairpin conformation of an otherwise flexible DNA strand during hairpin telomere formation by TelA.

### Refolding Intermediate

The crystal structures of the hairpin–DNA–TelA complexes suggest that a linear duplex substrate bound to the TelA dimer would be in a severely distorted conformation (Figure S3), as observed for the structure of TelK bound to a duplex substrate DNA [16], and is thus transformed into the thermodynamically more favorable hairpin form once the DNA strands are cleaved. Despite the overall favorable reaction energetics, refolding of a duplex substrate into two hairpin products within the TelA dimer may not readily proceed due to steric and/or electrostatic interferences. To gain insights into how the 5′-ends are exchanged, refolded, and packed tightly within the partially buried interior of the stable TelA dimer, we determined crystal structures of the covalent phosphotyrosine TelA–DNA intermediate trapped using suicide DNA substrates.

**Figure 7:**
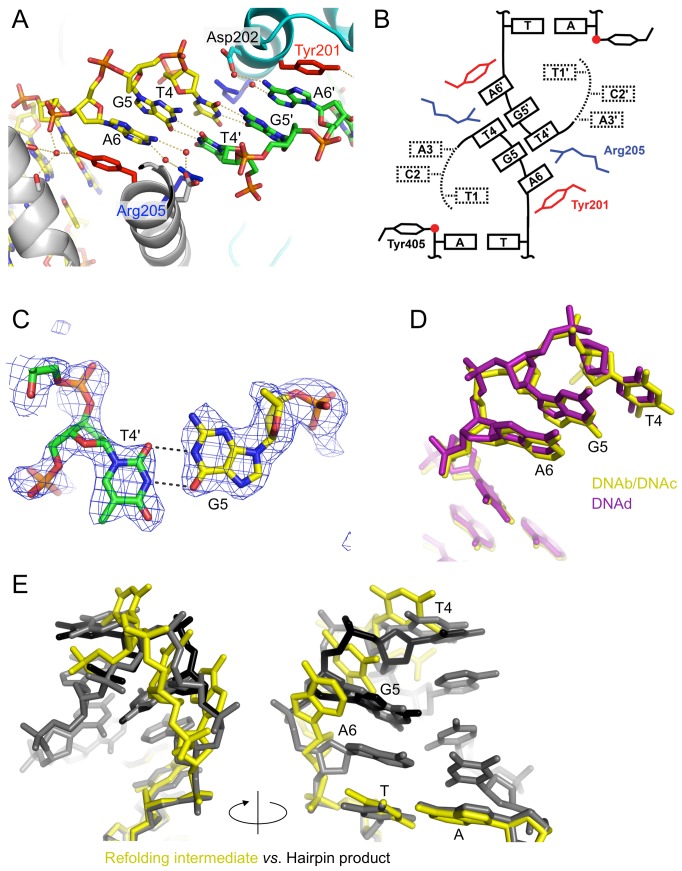
The strand-refolding intermediate. (A) The open DNA conformation observed in the phosphotyrosine complexes stabilized by stacking of the flipped-out bases, G-T wobble base-pairs, and water-mediated hydrogen bonds. The protein and DNA molecules are colored as in Figure 4A. (B) Schematic diagram of the strand-refolding intermediate DNA conformation. The 5′ bases Thy1, Cyt2, and Ade3 are flexible. Thy1 in our crystal structures is either missing or replaced by cytosine to block ligation and trap the phosphotyrosine bond. (C) Simulated annealing omit Fo-Fc electron density contoured at 3.0 σ for the G-T wobble pair. (D) 5′-overhang conformation observed in the phosphotyrosine complexes (the refolding intermediate) trapped using two different types of suicide DNA substrates. The structure obtained with the nicked suicide substrate (DNAb/DNAc) is shown in yellow, while that obtained with a mismatch-based suicide substrate (DNAd) is shown in purple. The tri-nucleotide stretch Thy4, Gua5, and Ade6 is superimposable with an r.m.s.d. of 0.78 Å. The deviation comes mostly from the phosphate backbone atoms. (E) Comparison of the DNA structures between the refolding intermediate (yellow) and the hairpin telomere product (alternate conformations shown in black and grey). The superposition is based on the double-stranded stem region of the DNAs.

The first type of suicide DNA substrate used in our studies has nicks one base 3′ to the scissile positions (DNAb or DNAc in Figure S1) [26]. Strand cleavage by TelA releases a single nucleotide (Thy1) between the nick and the newly formed phosphotyrosine bond, removing the 5′-OH group necessary for strand ligation. The resulting DNA has five-base 5′-overhangs, one base short of the natural six-base 5′-overhang. Crystal structure of this phosphotyrosine complex refined at 2.2 Å resolution (Figure 7A–C) shows a unique “open” conformation of the DNA strand distinct from that observed for the compact hairpin telomere product (Figure 7E). In this open conformation, the first base into the overhang region to be refolded (Ade6) completely swings out into an extrahelical position and stacks against the Tyr201 sidechain. The positioning of Tyr201 is determined by water-mediated hydrogen bonds with the main chain carbonyl group of Ile170, Thr174 sidechain, and a DNA backbone phosphate, whereas the swung-out conformation of Ade6 is stabilized by those involving Asp202 and Arg205 (Figure 7A). The base plane of Ade6 has a ∼45° tilt with respect to the base planes in the duplex region. The second base, Gua5, is also flipped out and is stacked on Ade6. The third base, Thy4, is positioned similarly to how it is positioned in the hairpin product structure (Figure 7E) where the Arg205 guanidinium group makes a cation-π stacking on its nucleobase moiety, while the other face of the Thy4 base stacks on its symmetry-mate across the 2-fold axis of the TelA complex. The sugar moiety of Thy4 stacks on the Gua5 base. In addition to these stacking interactions, the positioning of Gua5 and Thy4 is further stabilized by G-T wobble base-pairs (Figure 7A,C). The *syn* conformation of the Gua5 base is well supported by simulated annealing omit difference electron density (Figure 7C). While clear electron density was observed for the bases Thy4, Gua5, and Ade6, the density was weaker and less continuous for the bases Cyt2 and Ade3, suggesting higher flexibility near the 5′-terminus of the DNA strand (Figure S7).

**Figure 8:**
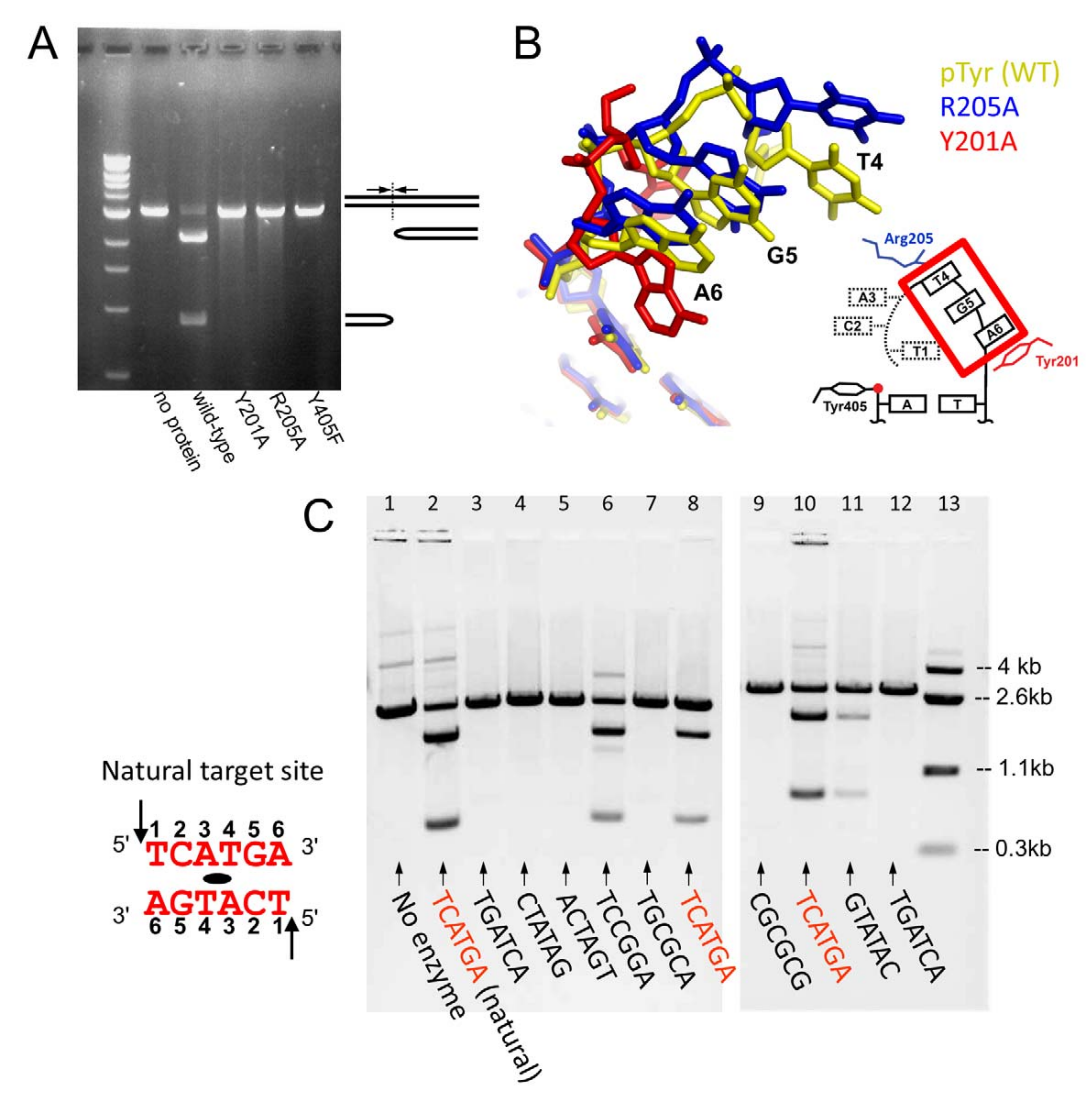
Requirements for the formation of hairpin products. (A) In vitro hairpin formation assay on the wild-type and mutant TelA proteins, showing that the non-active site residues Tyr201 and Arg205 are essential in hairpin product formation. Tyr405 is the catalytic nucleophile residue that forms the phosphotyrosine bond. (B) 5′-overhang conformations for mutant TelA–DNA complexes. The tri-nucleotide stretch including Thy4, Gua5, and Ade6 is shown for the refolding intermediate (phosphotyrosine complex formed with the wild-type TelA) in yellow, the DNA complexed with R205A in blue, and that complexed with Y201A in red. (C) In vitro hairpin formation by the wild-type TelA on DNA substrates with various 6 bp palindromic sequences between the scissile sites. Duplicated samples are with separate substrate DNA preparations.

In the open DNA conformation observed in the phosphotyrosine complex, the trajectory of the linearly stacked Tyr201 sidechain, Ade6, Gua5, and Thy4 bases is completely blocked by the interdomain α-helix harboring Arg205, and the DNA backbone makes a sharp turn to reverse the chain direction (Figure 7A,B and Figure S7). The irregular conformation of DNA with flipped-out bases, sharply bent backbone, and the flexible 5′-terminus suggests that it is an intermediate step in the DNA strand refolding pathway (Figure 1B). Stabilization of the flipped-out bases Ade6 and Gua5 in extrahelical positions would help disrupt the original base-pairing in the duplex substrate, as well as clear space for exchanging the 5′-ends. The path of the DNA strand is set by the stacking interaction made by Tyr201 that orients the first base Ade6 and the capping interaction made by Arg205 that shapes the nascent hairpin loop structure. Point mutants TelA•Y201A and TelA•R205A are capable of cleaving DNA to form the phosphotyrosine complex, but they are completely inactive in producing hairpin products in vitro (Figure 8A and Figure S8). This underscores the important roles of Tyr201 and Arg205 in refolding DNA into a hairpin telomere. Consistent with the critical role of Tyr201 in orienting the tri-nucleotide stretch Ade6, Gua5, and Thy4, a crystal structure of TelA•Y201A-DNA complex shows that Ade6 has swung out further toward the protein to partially occupy the space taken by Tyr201 in the wild-type enzyme, leaving other bases in the 5′-overhang flexible (Figure 8B). Similarly, a crystal structure of TelA•R205A complexed with DNA carrying the natural self-complementary six-base 5′-overhang (DNAb6 in Figure S1) shows an extended DNA conformation without hairpin loop formation or flipping of the Gua5 base into the *syn* conformation (Figure 8B).

Interestingly, Tyr319 and Arg322 of Tn5 transposase are part of a conserved “YREK” sequence motif found in the IS4 family of transposases and are involved in stabilizing the hairpin DNA conformation [18]. However, the precise roles of the Tyr and Arg sidechains appear to be different between Tyr319/Arg322 of Tn5 and Tyr201/Arg205 of TelA. Tyr201 of TelA rather plays an analogous role to Trp298 of Tn5 that stacks against a flipped-out DNA base in an extrahelical position [18],[27]. In any case, a critical difference between the transposon and the hairpin telomere systems is that while base-flipping occurs in the transposase-bound hairpin DNA [18],[28], it is observed only in the refolding intermediate (Figure 7A,B) and not in the hairpin telomere product (Figure 4A,B) bound to TelA.

The putative strand refolding intermediate with the open DNA conformation described above was obtained with a DNA substrate missing the terminal nucleotide Thy1 of the 5′-overhang (DNAb or DNAc in Figure S1). We reasoned that if it is indeed a transition state structure, the DNA strand could be trapped in the same conformation by introducing a mismatch in the natural palindromic six-base 5′-overhang to inhibit formation of the fully base-paired hairpin loop. Thus a second type of suicide DNA substrate was designed, in which the six-base overhang has cytosine in place of Thy1 to block base-pairing (DNAd in Figure S1). The Thy to Cyt substitution should also prevent the major groove interaction by Lys208 (Figure 6). Wild-type TelA mixed with this mismatched DNA substrate was trapped in a phosphotyrosine complex as expected. The crystal structure refined at 2.4 Å resolution shows that the six-base overhang adopts a conformation very similar to that observed for the nicked suicide substrate, with an r.m.s.d. of 0.78 Å for atoms in the tri-nucleotide stretch (Figure 7D). We therefore concluded that the open DNA conformation observed in the trapped phosphotyrosine complexes represents a metastable state of the 5′-overhang, which precedes stable capturing of the 5′-end into the fully base-paired hairpin form. We propose that this “refolding intermediate” conformation allows TelA to overcome steric and/or electrostatic interferences between the DNA strands during refolding of a duplex substrate into the compact hairpin products (Figure 1B). The mechanism is reminiscent of how enzymes in general catalyze chemical reactions by stabilizing intermediates, lowering energy barriers along reaction pathways [29].

### DNA Sequence Requirements

To further validate our model on the mechanism of hairpin telomere formation, we examined effects of sequence variation in the DNA substrate. Based on the observed sequence-specific interactions that stabilize the hairpin product or the refolding intermediate including the G-T wobble pair, one would expect a strict requirement in hairpin telomere formation for the specific 6 bp DNA sequence between the two scissile positions. We therefore tested in vitro hairpin formation by TelA on a series of duplex DNA substrates with various palindromic sequences in the central 6 bp (Figure 8C). Most of the nonnatural target sequences yielded no hairpin products, including 5′-TGATCA-3′ that corresponds to a natural target site after swapping Gua5 and Cyt2 (Figure 8C, Lanes 3 and 12). Nonetheless, these nonnative substrates were cleaved by TelA (unpublished data). The results are consistent with our model that the DNA strands are refolded into hairpins guided by a set of specific protein–DNA and DNA–DNA interactions. It would be fair to note, however, that there are nonnative sequences that still support in vitro hairpin formation (e.g., Figure 8C, Lane 6), and we found sequence changes at the tip of the hairpin turn (Ade3 and Thy4) to be more tolerated in general [5]. Alternative base-pairing schemes, a G–G base-pair for instance [30], may allow for formation of a refolding intermediate similar to that formed by the natural target sequence in such cases.

The fairly elaborate mechanism of strand refolding by TelA described in this work raises a question as to how well it is conserved among protelomerase enzymes from different organisms. The basic architecture of the protelomerase dimer with a sharp disjunction in the DNA-binding path has been observed for both TelA and the bacteriophage-derived TelK systems (Figure S3) [16] and is likely a common feature for this family of proteins. In addition, all known protelomerase enzymes make staggered DNA cleavages 6 bp apart [12],[25],[31], where the conserved spacing between the scissile phosphates may reflect similarities in general mechanisms of hairpin telomere formation. On the other hand, the amino acid residues of TelA outside its catalytic domain, including those important in DNA refolding (Tyr201 and Arg205), do not appear to be particularly well conserved among protelomerase enzymes from different systems [32]. Moreover, DNA sequences of the hairpin telomeres from different organisms do not show strong similarities and each protelomerase system has unique target sequences. We have shown that a modified palindromic DNA substrate with 5′-CGCGCG-3′ between the scissile positions, as found in the native target sequence for TelK [25], is not processed into hairpin products by TelA (Figure 8C, Lane 9). Similarly, TelK cleaves efficiently but does not produce hairpin products on a modified DNA substrate with 5′-GTATAC-3′, as found in the presumed target sequence for the protelomerase from phage VHML [16],[33]. The strict but distinct sequence requirements imply that stabilization of DNA refolding intermediates may be a common strategy employed by protelomerase enzymes, while the detailed refolding mechanisms, including specific protein–DNA and DNA–DNA interactions, likely differ among different organisms.

### Conclusion

Enzymes that rearrange/recombine DNA play important roles in diverse biological contexts [34]. A common strategy employed by these enzymes is to bind tightly to and stabilize the final products, thereby driving the reactions forward by virtue of DNA-binding free energies [35]–[37]. We have shown that this is indeed the case for the protelomerase TelA, a DNA rearrangement enzyme involved in the maintenance of *Agrobacterium* linear chromosome. Unexpectedly, however, we found that TelA facilitates the duplex-to-hairpin conversion by stabilizing not only the hairpin telomere product but also a transient strand-refolding intermediate to guide the DNA strand refolding process. We believe that the enzyme-catalyzed, multistep DNA refolding described in this study is a novel mechanism, and we suspect that similar strategies may be employed by other protein machineries that facilitate conformational changes/refolding of DNA or other macromolecules in various biological contexts.

## Materials and Methods

### Crystallization and Structure Determination

The TelA protein and its mutants used in the present study have a 20-residue N-terminal His-tag attached to the full-length *Agrobacterium tumefaciens* protelomerase protein (gene locus: Atu_2523). The His-tagged TelA was overexpressed in *E. coli* strain BL21 under the control of the arabinose-inducible pBAD promoter and purified using the Ni-NTA and Heparin-Sepharose columns. Selenomethionine-labeled TelA was overexpressed in the methionine auxotroph strain B834(DE3). All oligonucleotides were purchased from IDT in the standard desalting grade and used without further purification. The sequences of the oligonucleotides used in the crystallization experiments are available in Figure S1. The target sequence for TelA was derived from either the left terminus (for DNAa, DNAb, DNAd, and DNAb6 in Figure S1) or the right terminus (for DNAc used to grow SeMet crystals) of the *Agrobacterium tumefaciens* C58 linear chromosome (Genbank #AE007870.2).

TelA–DNA complexes used in crystallization were prepared by mixing equal moles of protein monomer and DNA half-site at an approximate protein concentration of 0.2 mM and a NaCl concentration of ∼0.5 M. The hairpin DNA–TelA–vanadate complex was assembled in the presence of 10 mM sodium orthovanadate using a DNA substrate containing the six-base TCATGA overhang (DNAa in Figure S1). The R255A TelA–hairpin DNA complex was prepared using the same DNA in the absence of sodium orthovanadate. To prepare the phosphotyrosine complexes with cleaved DNA, the purified protein was mixed with either the CATGA (nicked: DNAb, DNAc in Figure S1) or CCATGA (mismatched: DNAd in Figure S1) suicide DNA substrate. Crystals of the TelA–DNA complexes were obtained by the hanging drop vapor diffusion method at 20°C. The well solution consisted of 5% (w/v) PEG 4000, 10 mM Tris-HCl (pH 7.4), and 300 mM NaCl, and the hanging drops were formed by mixing the TelA–DNA complex with the well solution at a 1∶1 volume ratio. All TelA–DNA complexes were crystallized in the same crystal form under similar conditions, though the time it took for the crystals to appear varied depending on the DNA substrates used. The crystals were cryoprotected by gradually introducing glycerol to a final concentration to 25%, then flash frozen in liquid nitrogen.

X-ray diffraction data were collected at the beamlines 14ID-B, 14BM-C, and 24ID-C of the Advanced Photon Source (Argonne, IL). The structure of the TelA phosphotyrosine complex formed with a 1 bp longer suicide DNA substrate than the other DNA substrates (15 mer+19 mer: DNAc in Figure S1) was determined by the single-wavelength anomalous dispersion (SAD) method using the selenomethionine drivative. Two single-path scan datasets were collected at the selenium K-edge, one at a lower resolution (3.0 Å) and one to a higher resolution (2.4 Å). Indexing, integration, and scaling of the collected diffraction frames were done using HKL2000 [38] or XDS [39]. Eleven selenium sites per TelA monomer were located with the lower resolution dataset (3.0 Å) using SOLVE [40]. Resolution of the experimental phases was then improved by combining the two datasets, and density modification by RESOLVE generated an interpretable map. Automated model building by RESOLVE built ∼50% of the protein residues. Iteration of phase-restrained refinement using REFMAC5 [41] and manual model building using COOT [42] eventually generated a model consisting of 320 amino acids covering the TelA residues 102∼421, and all DNA residues. The 121 N-terminal amino acids including the 20-residue His-tag, and the 21 C-terminal amino acids are disordered. Structures of all other complexes were determined by molecular replacement using PHASER [43]. All crystallographic models were finally refined using PHENIX [44] with the TLS refinement. X-ray diffraction data, phasing, and model refinement statistics are summarized in Table 1. Severely anisotropic data were subjected to ellipsoidal truncation and anisotropic scaling [45] prior to structure refinements. Figures were produced using PYMOL (www.pymol.org). Buried protein surface area was calculated using CNS [46]. Curves [47] and 3DNA [48] were used for DNA geometry analyses.

### In Vitro Hairpin Formation and DNA Cleavage Assays

A 50 bp region from *Agrobacterium* chromosome terminus centered on the target sequence of TelA, or its variant sequences, was cloned into the pSK plasmid to generate pAgSK54. pAgSK54 linearized with the restriction enzyme AlwNI was purified using a Qiagen spin-column and was employed as the substrate in the in vitro resolution assay. The resolution reaction was performed with 10 nM linearized plasmid DNA and 6.7 µM TelA at 22°C for 2 h in 50 mM Tris-HCl (pH 7.4), 150 mM NaCl, and 1 mM DTT (Figure 8A) or at 30°C for 30 min in 20 mM Tris-HCl (pH 7.5), 50 mM potassium glutamate, and 1 mM DTT (Figure 8C). The reactions were quenched by addition of proteinase K or SDS. The DNA products extracted by phenol extraction were separated on 1% argarose gel and visualized by ethidium bromide staining. The phosphotyrosine complex formation of the wild-type and mutant TelA proteins (Figure S8) was monitored on a 44 bp nicked suicide substrate assembled from 24 mer and 20 mer oligonucleotides containing the 5′-CATG-3′ overhang. Protein at 10 µM was incubated with 2× molar excess of the suicide substrate DNA in the same buffer condition used for the resolution reaction. The reaction was quenched at each time point (1 h, 2 h, 1 d, 2 d, 3 d, and 1 wk) by addition of SDS and the samples were analyzed by SDS-PAGE.

### Accession Codes

The atomic coordinates and the structure factors for all crystal structures reported here have been deposited in the Protein Data Bank with accession codes 4E0G, 4E0J, 4E0P, 4DWP, 4E10, 4E0Z, and 4E0Y.

## Supporting Information

Figure S1 DNA substrates used in the crystallographic studies. Each substrate was assembled from two oligonucleotides of different lengths to form a 5′-overhang. The red letters correspond to the region between the two scissile phosphates. DNAc is based on the chromosome right terminal sequence, whereas all others are based on the left terminal sequence shown at the top. The differences between the left and right telomere sequences are limited to the positions highlighted by the green boxes.
(TIF)

Figure S2 Comparison between *Agrobacterium tumefaciens* TelA and the protelomerase TelK from bacteriophage ΦKO2. (Top) Superposition of the two crystal structures (DNA was omitted for clarity). TelK has a large insertion (muzzle) within the α-helical bundle domain opposite the catalytic domain and an additional DNA-binding domain in the C-terminus (stirrup) [16], both of which are absent from TelA. (Bottom) Amino acid sequence alignment for the region of the catalytic domain harboring the active site residues. TelA residues highlighted by the colored boxes are Arg255, Lys286, Tyr363, Arg366, His394, and Tyr405.
(TIF)

Figure S3 Disjunction in the DNA helical axis observed in the TelA–DNA and TelK–DNA complexes. The helical axes of the two hairpin DNA products bound to the TelA dimer (bottom) have a large (>10 Å) offset. The TelK–DNA complex (top) was reported to have a similar arrangement, with ∼7.5 Å offset in the DNA axis across the dimer interface [16]. The TelA–DNA and TelK–DNA complexes are shown in the same orientation (viewed along the 2-fold axis from the catalytic domain side).
(TIF)

Figure S4 An overview of sequence-specific (as well as some backbone) DNA interactions made by TelA.
(TIF)

Figure S5 Water-mediated interactions in the sequence recognition. Water-mediated hydrogen-bonds involved in the DNA sequence recognition by TelA are highlighted by dotted lines. 2Fo-Fc electron density is contoured at 1.5 σ. Water molecules are shown by the red spheres.
(TIF)

Figure S6 Molecular packing in the TelA–DNA complex crystals. Sections through the crystal lattice parallel to different faces of the unit cell. A pair of blue and red TelA molecules corresponds to the biologically relevant dimer responsible for resolving the replicated hairpin telomere sequence. There is no lattice contact between the TelA dimer–DNA complexes around the hairpin DNA termini located in the middle of the complex.
(TIF)

Figure S7 Flexibility of the DNA strands in the refolding intermediate. Simulated annealing composite omit 2Fo-Fc map contoured at 1.5 σ for the central region of the TelA phosphotyrosine complex (strand refolding intermediate conformation). Patchy density for the 5′-terminal nucleotides (not present in the atomic model; drawn with dotted lines in the cartoon) suggests high flexibility. A wall-eye stereo pair is shown.
(TIF)

Figure S8 In vitro DNA cleavage assays for the wild-type and mutant TelA proteins, showing that Tyr201 and Arg205 are not essential for DNA cutting (phosphotyrosine bond formation).
(TIF)

## Abbreviations

bp: base-pair
DNA: deoxyribonucleic acid
DTT: dithiothreitol
FOM: figure of merit
PAGE: polyacrylamide gel electrophoresis
PEG: polyethylene glycol
p-Tyr: phosphotyrosine
r.m.s.d.: root-mean-square deviation
SDS: sodium dodecyl sulfate
TLS: translation-libration-screw
Tris: tris(hydroxymethyl)aminomethane
