# Supplementary figures and images for: An Enzyme-Catalyzed Multistep DNA Refolding Mechanism in Hairpin Telomere Formation
Source: PLoS Biol. 2013 Jan 29;11(1):e1001472. doi: 10.1371/journal.pbio.1001472 (PMC3558466; doi:10.1371/journal.pbio.1001472)

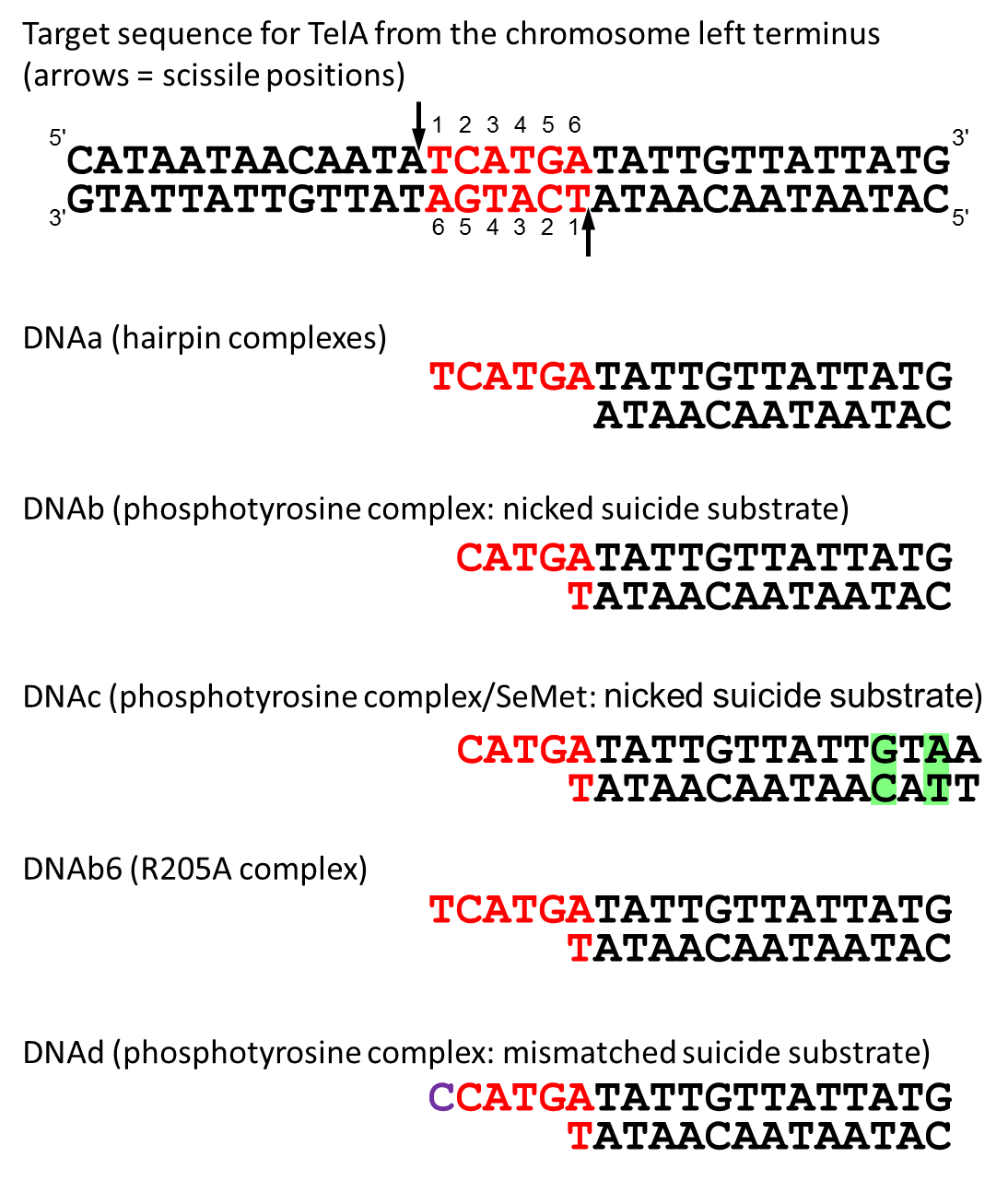

Supplement: Figure S1 — DNA substrates used in the crystallographic studies. Each substrate was assembled from two oligonucleotides of different lengths to form a 5′-overhang. The red letters correspond to the region between the two scissile phosphates. DNAc is based on the chromosome right terminal sequence, whereas all others are based on the left terminal sequence shown at the top. The differences between the left and right telomere sequences are limited to the positions highlighted by the green boxes. (TIF) [file pbio.1001472.s001.tif]

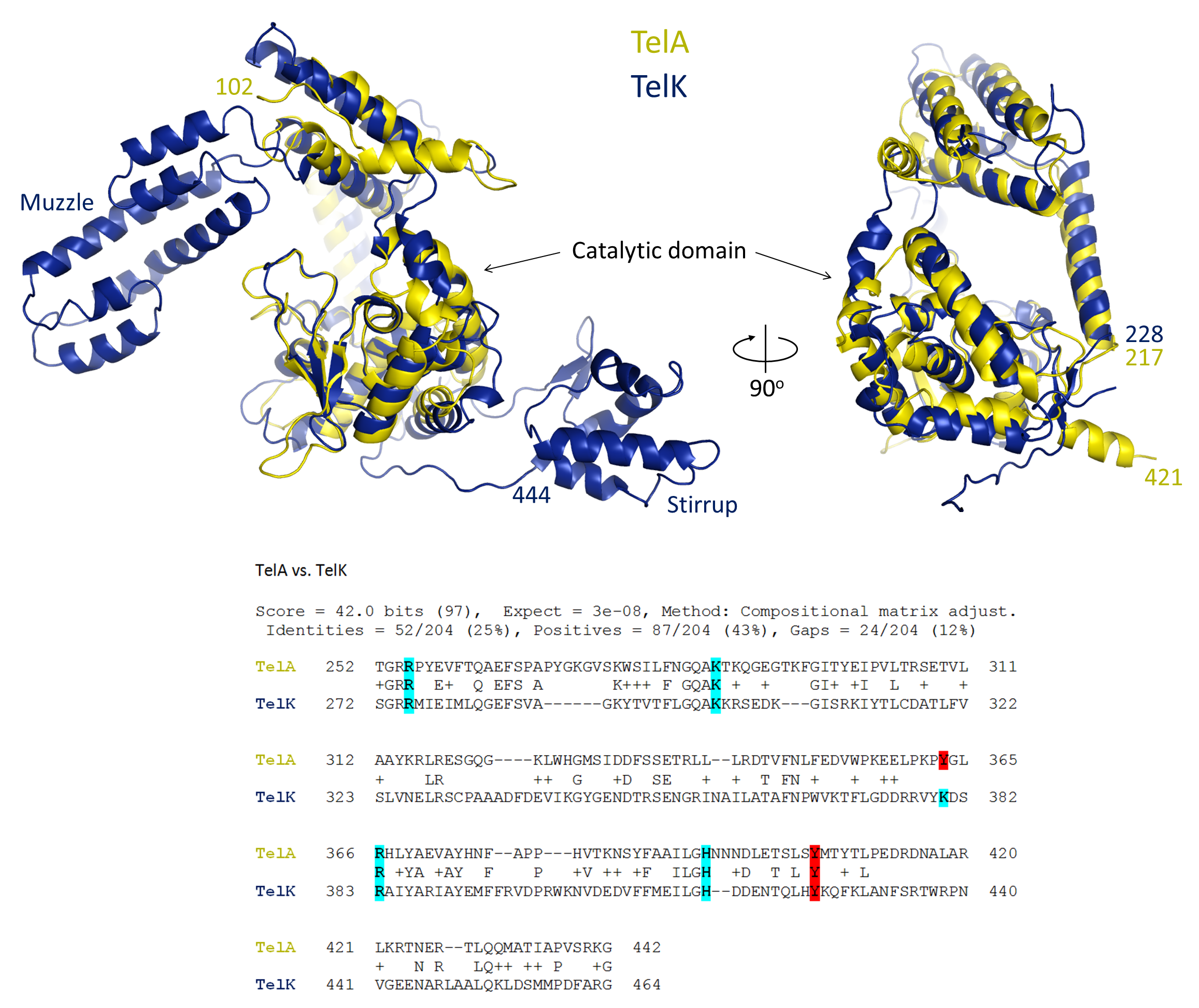

Supplement: Figure S2 — Comparison between Agrobacterium tumefaciens TelA and the protelomerase TelK from bacteriophage ΦKO2. (Top) Superposition of the two crystal structures (DNA was omitted for clarity). TelK has a large insertion (muzzle) within the α-helical bundle domain opposite the catalytic domain and an additional DNA-binding domain in the C-terminus (stirrup) [16], both of which are absent from TelA. (Bottom) Amino acid sequence alignment for the region of the catalytic domain harboring the active site residues. TelA residues highlighted by the colored boxes are Arg255, Lys286, Tyr363, Arg366, His394, and Tyr405. (TIF) [file pbio.1001472.s002.tif]

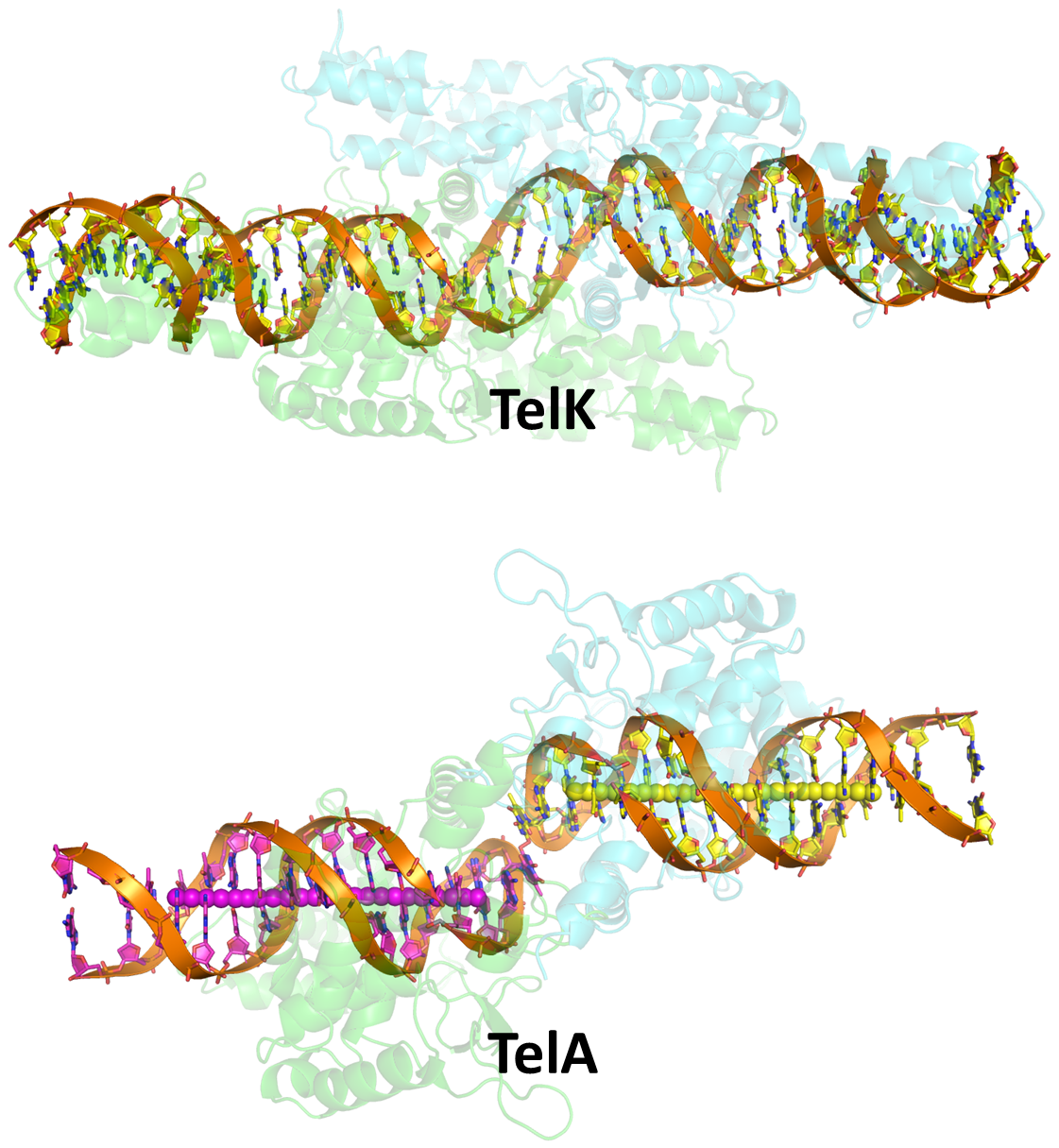

Supplement: Figure S3 — Disjunction in the DNA helical axis observed in the TelA–DNA and TelK–DNA complexes. The helical axes of the two hairpin DNA products bound to the TelA dimer (bottom) have a large (>10 Å) offset. The TelK–DNA complex (top) was reported to have a similar arrangement, with ∼7.5 Å offset in the DNA axis across the dimer interface [16]. The TelA–DNA and TelK–DNA complexes are shown in the same orientation (viewed along the 2-fold axis from the catalytic domain side). (TIF) [file pbio.1001472.s003.tif]

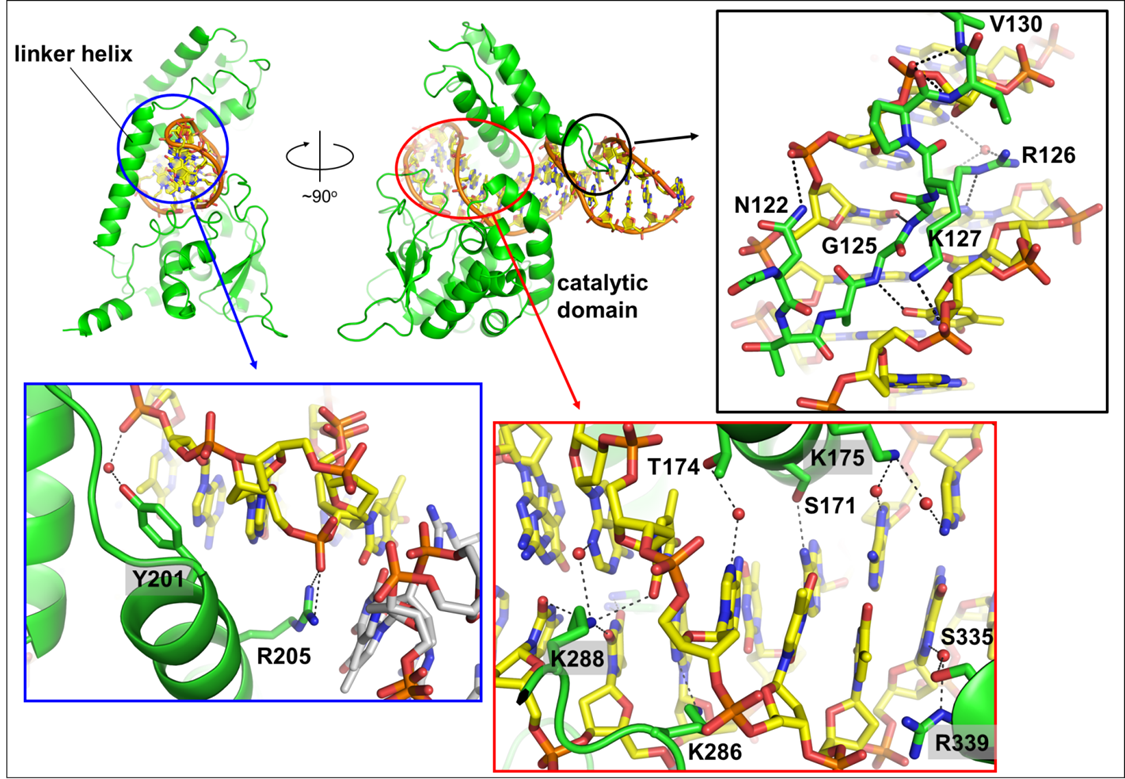

Supplement: Figure S4 — An overview of sequence-specific (as well as some backbone) DNA interactions made by TelA. (TIF) [file pbio.1001472.s004.tif]

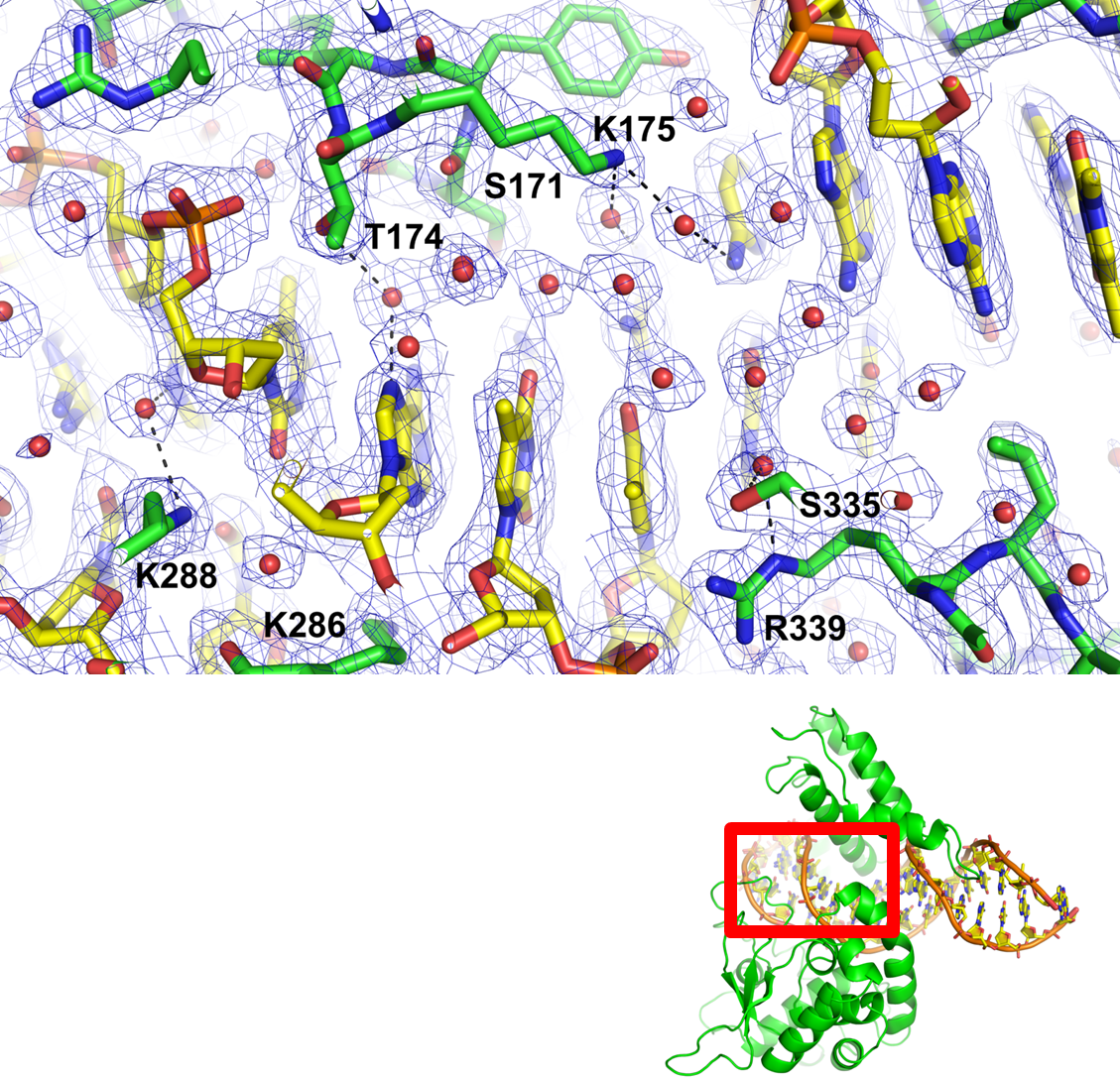

Supplement: Figure S5 — Water-mediated interactions in the sequence recognition. Water-mediated hydrogen-bonds involved in the DNA sequence recognition by TelA are highlighted by dotted lines. 2Fo-Fc electron density is contoured at 1.5 σ. Water molecules are shown by the red spheres. (TIF) [file pbio.1001472.s005.tif]

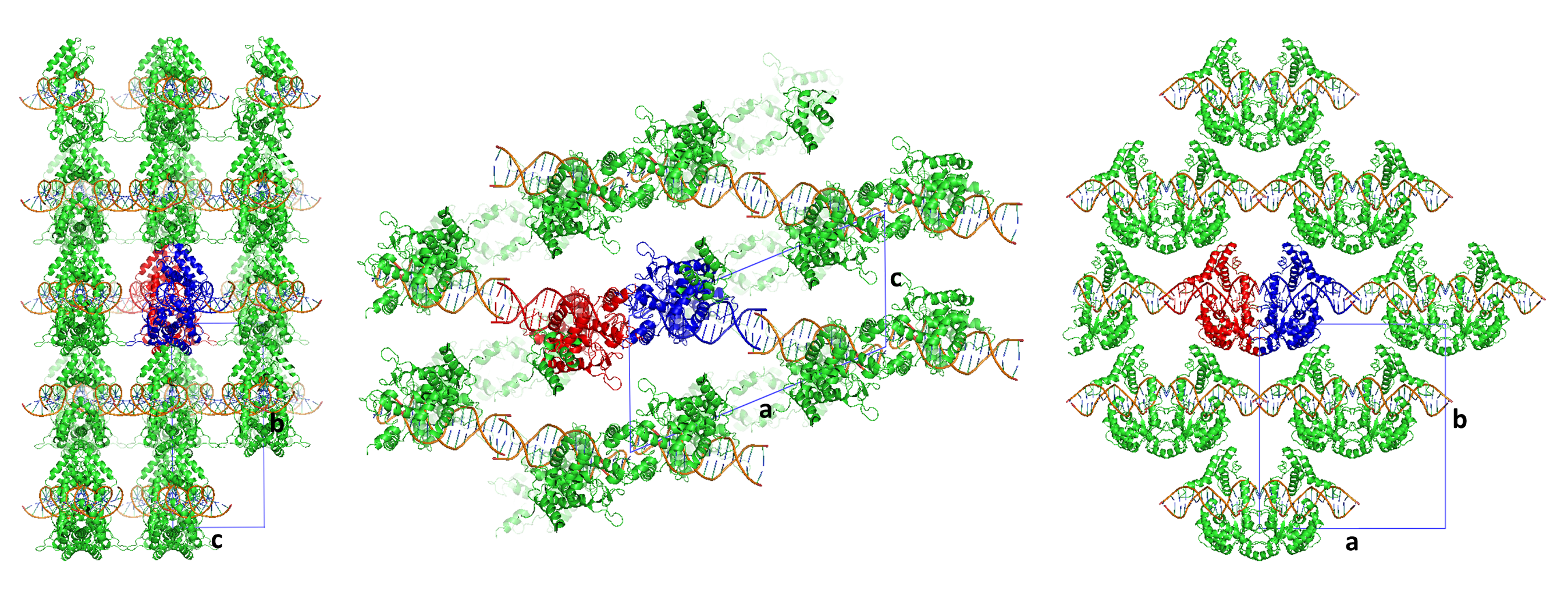

Supplement: Figure S6 — Molecular packing in the TelA–DNA complex crystals. Sections through the crystal lattice parallel to different faces of the unit cell. A pair of blue and red TelA molecules corresponds to the biologically relevant dimer responsible for resolving the replicated hairpin telomere sequence. There is no lattice contact between the TelA dimer–DNA complexes around the hairpin DNA termini located in the middle of the complex. (TIF) [file pbio.1001472.s006.tif]

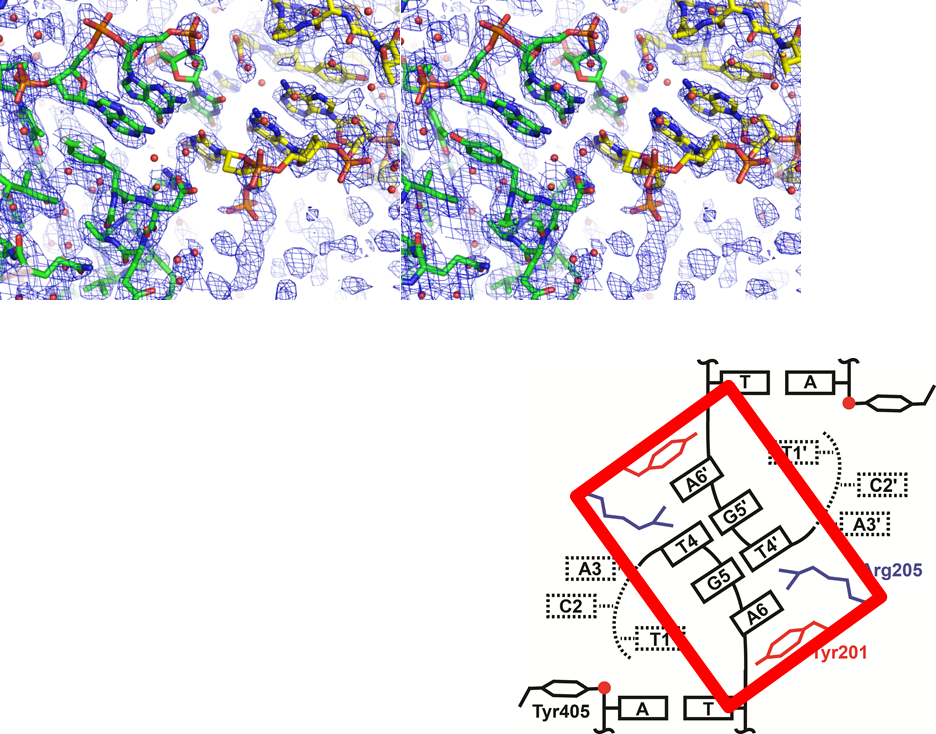

Supplement: Figure S7 — Flexibility of the DNA strands in the refolding intermediate. Simulated annealing composite omit 2Fo-Fc map contoured at 1.5 σ for the central region of the TelA phosphotyrosine complex (strand refolding intermediate conformation). Patchy density for the 5′-terminal nucleotides (not present in the atomic model; drawn with dotted lines in the cartoon) suggests high flexibility. A wall-eye stereo pair is shown. (TIF) [file pbio.1001472.s007.tif]

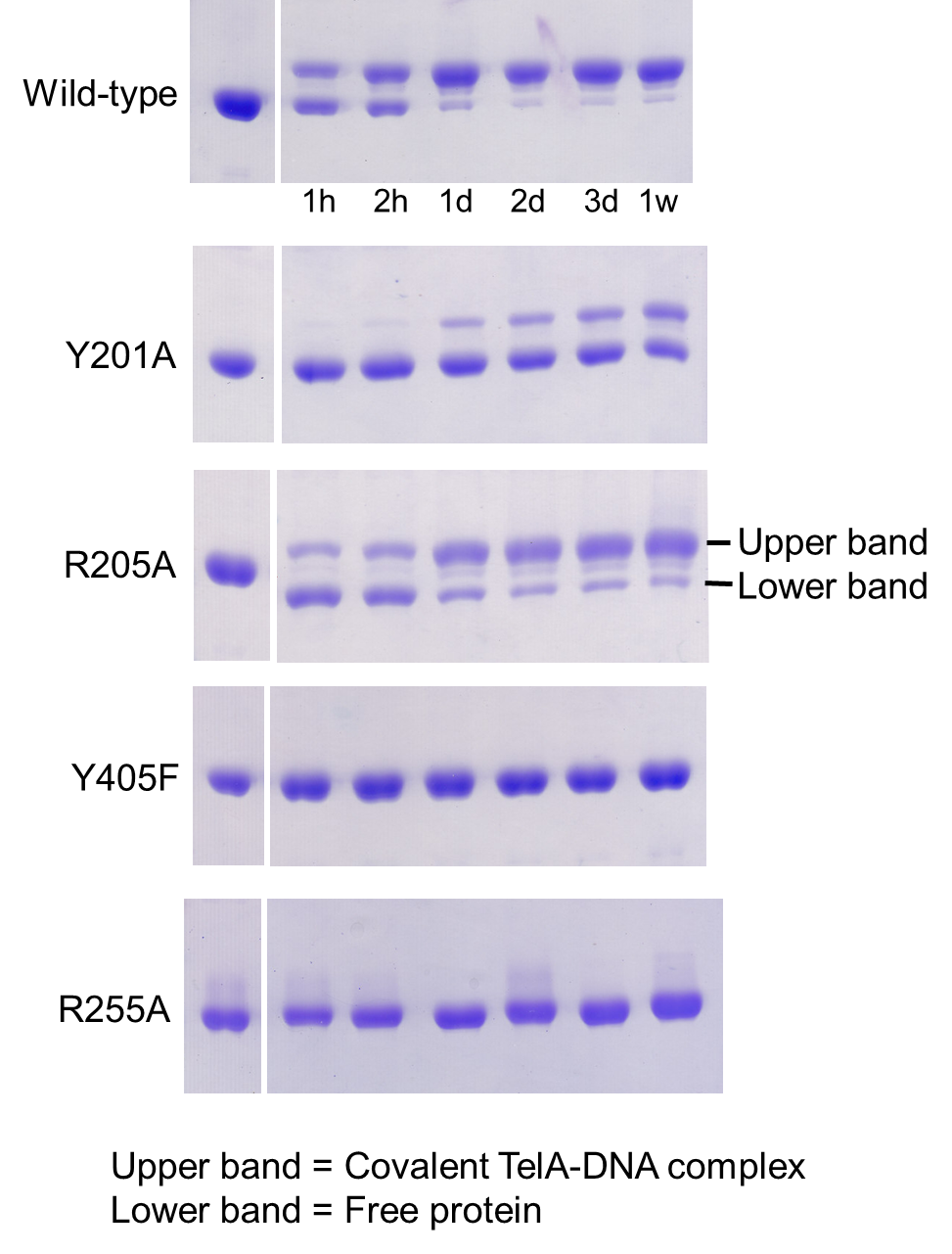

Supplement: Figure S8 — In vitro DNA cleavage assays for the wild-type and mutant TelA proteins, showing that Tyr201 and Arg205 are not essential for DNA cutting (phosphotyrosine bond formation). (TIF) [file pbio.1001472.s008.tif]
